# Clinician Perspectives on Commonly Used Online Sexual and Reproductive Health Resources for Adolescents: Qualitative Analysis

**DOI:** 10.2196/89643

**Published:** 2026-04-23

**Authors:** Cambray Smith, Sarah Rebbeor, Elizabeth Pleasants, Leah Frerichs, Bianca A Allison

**Affiliations:** 1 Department of Health Policy and Management, Gillings School of Global Public Health, University of North Carolina at Chapel Hill, 135 Dauer Dr., 1101 McGavran-Greenberg Hall CB #7411, Chapel Hill, NC, 27516, United States; 2 Medical Scientist Training Program, School of Medicine, University of North Carolina at Chapel Hill, Chapel Hill, NC, United States; 3 School of Medicine, University of North Carolina at Chapel Hill, Chapel Hill, NC, United States; 4 Center for Women's Health Research, School of Medicine, University of North Carolina at Chapel Hill, Chapel Hill, NC, United States; 5 Department of Pediatrics, School of Medicine, University of North Carolina at Chapel Hill, Chapel Hill, NC, United States

**Keywords:** adolescent, sexual and reproductive health, digital literacy, social media, health literacy

## Abstract

In this interview study with 24 adolescent-serving clinicians, participants described current online sexual and reproductive health resources they share with adolescents and highlighted areas of improvement to better meet the developmental needs of this age group.

## Introduction

Adolescents frequently search for and encounter information about sexual and reproductive health (SRH) online [1,2]. While some of this information may be high-quality, patients also access misleading, inaccurate information that can negatively impact health outcomes, with long-term implications [3,4].

Clinicians depend on reliable, accessible online resources to support effective patient education and engagement; however, they may be unfamiliar with peer-recommended websites. The objective of this paper was to identify commonly used online SRH resources, examine expert-identified key considerations for adolescent populations, and assess potential gaps in existing materials to inform the development of improved resources.

## Methods

### Overview

These findings are part of a broader qualitative study focused on improving clinician-adolescent communication about online information [5]. Self-identified adolescent SRH experts providing clinical care in the United States were recruited through national listserves (Society for Adolescent Health and Medicine, Society of Family Planning) to participate in audio-recorded semistructured interviews over videoconferencing. Clinicians were asked the following questions embedded within a larger interview guide: “Are there any online resources that you provide adolescent patients about SRH? What are resources that you wish you had?”

Transcripts were audited for accuracy, and data were analyzed via the matrix method through rapid qualitative analysis [6] by two team members, with careful review by three additional team members. Participants were provided an opportunity to review and provide feedback on the synthesized findings.

### Ethical Considerations

This study was designated exempt by the University of North Carolina at Chapel Hill Institutional Review Board (#24‐2060). All participants provided informed consent through an electronic survey prior to participation. Data were stored in password-protected files accessible only to the research team. Participants were compensated with a US $50 gift card for their time.

## Results

### Participant Characteristics

Overall, 24 clinicians participated in our study, representing all US regions. Most were White (n=18, 75%), cisgender women (n=19, 79%), and physicians (n=20, 83%) who were trained in pediatrics (n=17, 71%) and practiced in academic settings (n=20, 83%; Multimedia Appendix 1).

### Common Resources

Clinicians described using 10 online sources for high-quality patient-facing information, with various considerations shared for each (Table 1). Many clinicians described being comfortable using 1 to 2 specific resources, and some discussed finding it challenging to keep up with new options. The most recommended patient-facing websites were Bedsider and the Center for Young Women’s/Men’s Health from Boston Children’s Hospital, although several participants cautioned that Bedsider may be most appropriate for older adolescents and young adults.

**Table 1.** Clinician-recommended sexual and reproductive health (SRH) information sources with considerations for adolescent populations.

Resource name, description, and social media handle (if applicable)	Reasons to recommend the resource	Limitations or specific patient considerations
Amaze: Provides an overview of developmentally appropriate SRH topics through animated videos (@amazeorg)	Patient-friendly content that may be especially appropriate for younger adolescents Corresponding parent content Available in many languages	May be less relevant for older adolescent populations
Bedsider: An online resource focused on contraception, abortion, relationships, and health sexuality (@bedsider)	Patient stories Interactive tools Consistent social media presence	May be more focused on sex positivity versus medical management Parents may find content inappropriate for younger adolescents
Centers for Disease Control and Prevention: Overview of information about contraception and STIs^a^, including options like pre-exposure prophylaxis	Up-to-date materials across SRH topics Familiar to most clinicians Standardized recommendations and guidelines	Some clinicians concerned about current accuracy and availability of SRH information due to administrative changes
Go Ask Alice: Interactive website with question-and-answer format, offered through Columbia University	Interactive and provides direct answers to specific questions	Not described beyond concerns related to the responsibility of clinicians to provide accurate information and being concerned about offline complications after following online advice
Individual clinician social media content creators (eg, @drjenniferlincoln, @drjengunter)	Allows adolescents to access information on the platforms they already use Informal education and more engaging content formats May be targeted to specific demographic or cultural groups	Hard for clinicians to verify content and may not feel comfortable recommending content that they have not seen May prefer recommending institutional or organizational social media content
Individual institutional resources: Format varies by institution (eg, websites, brochures/pamphlets, social media accounts)	May be perceived as more trustworthy depending on patient/family relationship with the institution Can offer local service recommendations	May be redundant if other institutions are providing similar resources More upkeep Institutional social media often more focused on parents
Planned Parenthood: Provides local clinical services and online educational content (@plannedparenthood)	Site covers a variety of sexual and reproductive health topics (eg, contraception, STIs) Can provide local recommendations for how to access care	Political baggage and may be concerning to parents (ie, assumed connection to abortion)
Reproductive Health Access Project: Online patient-friendly guides for different forms of contraception and abortion care (@reproductiveaccess)	Nongendered user guides and fact sheets Based on patient-centered values (ie, not just efficacy)	Not described
Scarleteen: Sexual education site focused on queer identities (@scarleteenorg)	Interactivity LGBTQ+^b^ friendly information Engagement on social media	Not described
Young Women’s and Young Men’s Health: Online health resource pages managed by Boston Children’s Hospital (@bch_cywh for Young Women’s Health)	High-quality, comprehensive health information across a variety of topics (including beyond SRH) Available in Spanish	Gendered name may make site feel less applicable to gender minority youth Fewer interactive features (ie, more reading)

a STI: sexually transmitted infection.

b LGBTQ+: lesbian, gay, bisexual, transgender, queer/questioning, plus (others).

#### Considerations When Sharing Resources

Clinicians stressed the importance of matching resources to the developmental stage of patients, their general literacy skills, and cultural background (Table 2). A few explained that they did not want to overload patients by sharing too many options. Clinicians also discussed the importance of considering the role of parents when administering resources, describing three common scenarios. First, some described providing joint educational resources for adolescents and parents to review together (eg, if a patient was considering contraception to manage heavy menstrual bleeding). Second, some clinicians provided direct parent-facing resources to help support the child’s pubertal development and discussion of common SRH topics. Finally, some clinicians described being cautious in situations where parents may be monitoring adolescents’ internet use without their child’s knowledge (eg, through search histories), prompting potential concerns about certain content recommended to adolescents (eg, political baggage with Planned Parenthood).

**Table 2.** Current and future considerations when recommending sexual and reproductive health (SRH) resources to adolescent patients.

Themes and subthemes	Illustrative quotations
Considerations when recommending online SRH resources	
Developmental stage	“For things like Bedsider, it was really designed for 21- to 29-year-olds. It’s very sex positive, like the Frisky Friday emails and things that are for people who are very comfortable with their sexuality and concepts around sexual health. I think it resonates a lot less with people who are earlier in their journey, or are in cultural communities for which sex positivity isn’t the way that this is framed... So I want I want it to be somewhat values-aligned.” (Participant 8)
Cultural relevance	“But there’s another [clinician influencer that I recommend] that talks a lot about PCOS and sexual health. And she is actually Muslim. And it’s a nice perspective to see that from a Muslim healthcare provider [since I care for] a large Muslim population with my patients.” (Participant 15)
Literacy skills and accessibility of information	“But a lot of my patients don’t really like to read, or they don’t have good reading skills, or they don’t read well in English, and they’d rather watch something. They’d rather listen to something and watch something at the same time, or they prefer to be presented in like a case vignette style of a story or in shorter clips or shorter amounts of information... They can watch, listen, interact, get feedback.” (Participant 18) “We give Bedsider and Young Women’s Health to every single person with a uterus that comes in a clinic because they’re accessible and have such good information.” (Participant 11)
Parental supervision or content directed to parents	“I hear from patients not uncommonly that they can’t look at websites like Planned Parenthood, because if their parents saw a Planned Parenthood in a search engine history or on a web browser, they would freak out because of the historical baggage that comes with the name Planned Parenthood. I don’t ever talk about it in [state name].” (Participant 4) “Amaze, literally at the top, has a whole parent section, and so I think that they acknowledge that parents will be on the website. They expect it, and they’re not trying to hide anything. And so that’s why I really like that.” (Participant 4)
Desired qualities in future online resources	
Adolescent codevelopment and guidance	“I think that it is important to have adolescents and young adults involved... I think that it’s very clear to me that I’m no longer an adolescent, and so like I don’t know where the spaces are that this information will be accessed, where [are] the most relevant places people are going and how people are searching. We can hypothesize all we want, but I think that if we really want to create resources and put them in places, the adolescents are on the internet and social media. We need to engage that traffic in those decisions. Maybe I would Google my institution and that’s how I would get to the resource, but that might not be how an adolescent is doing it.” (Participant 24) “Yeah, I think something that engaged patients to create it and not healthcare providers. I mean, we could be involved. But we really need patients and young people to explain how to explain this to their peers... So, for example, for the birth control sheets that we came up with like in [state name] we engaged young people, and it turned out talking about menstrual cycles was horrible. They were like, ‘What...is a menstrual cycle?’ and we were like, ‘Oh, it’s your period.’ And even the word contraception was hard. They were like, ‘Can you just say birth control?’” (Participant 4)
Availability in languages other than English	“I think one of the downfalls of a lot of resources, especially for my patients, is that are that they’re not available in languages really other than Spanish and so it doesn’t matter if I’m pointing to the best information in the world...[if] they can’t access it or read it.” (Participant 25)
Established organizations putting content on social media	“We have a resource sheet and I remember putting it together and being like, ‘Would I really use this in clinic?’... When I stopped to think about it from more of a research perspective, I was like, ‘I don’t think people are going to use this.’ And I didn’t want it to just be a thing that nobody used. I think that’s what made me think about like, maybe Bedsider is a good example. They have an Instagram page, they have a TikTok handle, so why am I giving them the website when they’re not going to go to a website?” (Participant 22)
Knowledge about individual creators making high-quality content	“I know that there are a number of great OB/Gyns, for example, that do a lot of reproductive health. That can be great. I know a lot of institutions now are trying to have representatives send out information in different contexts with a little bit more scientific rigor ideally... I know there are definitely a ton of other providers out there who are making good evidence-based content. But I think the question is can we take part of that, and then combine it with other sources?” (Participant 3) “I just started doing a dive...on some of the information available on social media related to abortion. And so I haven’t yet incorporated giving Instagram accounts or TikTok accounts to patients, but I’m thinking about that, as we did in a deep dive, and what might be coming up for folks, and so wondering if there’s room to say, ‘Oh, this is an account I suggest you follow, or something like that, and something to consider.’” (Participant 24) “If I were more of an active social media user and I knew the content creators out there that I think are like really nailing this like I would definitely recommend specific people.” (Participant 8)
Engaging, interactive format versus static, text-based resources	“People really don’t like static resources anymore. And this is subjective, but from doing trainings and hearing from people and then dealing with students, something that’s like, ‘Here’s a website that lays out a bunch of text and tells you about stuff,’ I don’t think it speaks to people anymore. So I think...videos and interactives, and it’s real people, and it’s not necessarily like an explanation of something, but it’s storytelling, I think, is helpful for people. Interactives where people can enter—like decision aids kinds of things, like ‘These are the side effects that would bother me,’ people are able to interact with and personalize things. That trend toward personalization is very widespread people want things that are tailored to them.” (Participant 13)
Patient/family-facing centralized location	“I think having a more centralized website available. There’s bits and pieces of every website that I can pick from knowing what the complaint is, and I can do that as a physician. But I think for patients and parents to be able to go to is the biggest bang. I don’t think those are readily available.” (Participant 25)

### Improvements for Future Resources

Participants expressed a desire for more resources to be available in languages other than English (Table 2). Some described challenges with information on social media being more engaging and accessible than resources created by health care institutions, making it less likely that patients would visit recommended websites than use social media resources. Accordingly, clinicians often wished for specific social media content to recommend, although a few described concerns about this type of resource recommendation since they did not feel that they were able to vet all information from individual creators. Social media accounts from health care institutions were sometimes seen as a good way to combine these goals, although clinicians often described these as currently being aimed at parents as opposed to adolescents.

In general, participants recommended that future resources be created or revised in partnership with adolescents who can help ensure that the resources are developmentally appropriate and engaging, and use patient-friendly language. They also recommended posting these resources in a centralized location that can be updated regularly by experts, which they described would be especially helpful for general pediatricians who may be less familiar with adolescent SRH, as well as for adolescents and parents.

## Discussion

Clinicians recommended a variety of online SRH resources to refer adolescents to outside of the clinical encounter. Resources had pros and cons, with certain sources being more appropriate for different developmental stages. Importantly, this study was not exclusively focused on generating a list of online resources—thus, this may not be an exhaustive list of adolescent-focused SRH online resources—nor does it comprehensively cover all factors that may impact individualized resource provision. Other limitations include a relatively small and homogeneous clinician sample and data collection embedded in a larger study, both of which may reduce the diversity of resources discussed.

Future work is needed for adolescent-engaged content development [7], resources available in languages other than English [8], and integration of high-quality medical content into commonly used platforms (eg, social media) to more effectively reach patient populations who often encounter health information on social networking sites [9]. Additionally, while the Society for Adolescent Health and Medicine currently has a website where many of these resources are compiled, it does not include some of the considerations mentioned by participants (eg, parental concerns) that may impact individualized resource provision [10]. Creating an up-to-date, detailed resource database that is easily accessible to patients, families, and clinicians—potentially supplemented by a list of recommended social media accounts to follow—is one way to help bridge this gap.

These findings can be used to improve identification of developmentally relevant and accurate online SRH content—as well as creation of new resources—that can improve adolescent well-being amid a complex digital information environment.

## Supplementary material

10.2196/89643 Multimedia Appendix 1 Participant characteristics of 24 adolescent-serving clinicians.

## Abbreviations

SRH: sexual and reproductive health
