# Supplementary material for: Clinician Perspectives on Commonly Used Online Sexual and Reproductive Health Resources for Adolescents: Qualitative Analysis
Source: JMIR Pediatr Parent. 2026 Apr 23;9:e89643. doi: 10.2196/89643 (PMC13105424; doi:10.2196/89643)
Supplement: Multimedia Appendix 1 [file pediatrics-v9-e89643-s001.docx]

**Multimedia Appendix 1. Participant characteristics of 24 adolescent-serving clinicians**

| **Characteristic** | **n/%**^a^  **N=24** |
| --- | --- |
| Gender |  |
| Cisgender man | 5 (21%) |
| Cisgender woman | 19 (79%) |
| Age |  |
| Mean (SD) | 41.5 (8.9) |
| Missing | 2 |
| Race and ethnicity^b^ |  |
| Asian | 1 (4%) |
| Black/African American | 2 (8%) |
| Hispanic/Latine | 4 (17%) |
| White | 18 (75%) |
| Other identities not listed | 2 (8%) |
| Sexual orientation |  |
| LGBQ+ | 5 (21%) |
| Heterosexual or Straight | 19 (79%) |
| Clinical role |  |
| Nurse Practitioner (NP) or Midwife (MSN) | 4 (17%) |
| Physician (MD/DO) | 20 (83%) |
| Specialty |  |
| Family Medicine | 3 (13%) |
| OB/Gyn^c^ | 4 (17%) |
| Pediatrics or Med-Peds | 17 (71%) |
| Adolescent medicine fellowship (MD/DO only, excluding OB/Gyns n=17) | 16 (94%) |
| Years of practice since completing residency |  |
| Mean (SD) | 10.5 (9.3) |
| Median [Min, Max] | 3, 37 |
| Region |  |
| Midwest | 7 (29%) |
| Northeast | 8 (33%) |
| South or Southeast | 4 (17%) |
| West | 5 (21%) |
| Practice type |  |
| Academic medical center | 20 (83%) |
| Community setting^d^ | 5 (21%) |
| Other | 1 (4%) |
| Hours/week of clinical care |  |
| Less than 10 hours | 6 (25%) |
| 10-20 hours | 6 (25%) |
| 21-30 hours | 5 (21%) |
| More than 30 hours | 7 (29%) |
| Number of teen patients seen a week |  |
| Fewer than 5 | 4 (17%) |
| 6-10 | 4 (17%) |
| 11-15 | 2 (8%) |
| More than 16 | 14 (58%) |
| Social media use |  |
| Multiple times a day | 9 (38%) |
| Daily | 8 (33%) |
| Multiple times a week | 3 (13%) |
| Weekly | 2 (8%) |
| Never | 2 (8%) |
| Self-categorized expertise |  |
| Adolescent sexual and reproductive health | 24 (100%) |
| Patient-centered communication about online health information | 8 (33%) |
| Pediatric digital wellness | 7 (29%) |

^a^Percentages may not add to 100 due to rounding or because of multiple-selection answers.

^b^Participants were asked in a single question to select the racial and ethnic identities with which they identify.

^c^Sub-specialties included pediatric and adolescent gynecology and complex family planning.

^d^Includes school-based health centers.
